# Switches in food and beverage product purchases can reduce greenhouse gas emissions in Australia

**DOI:** 10.1038/s43016-024-00971-6

**Published:** 2024-05-28

**Authors:** Allison Gaines, Maria Shahid, Daisy Coyle, Eden Barrett, Michalis Hadjikakou, Jason H. Y. Wu, Fraser Taylor, Simone Pettigrew, Bruce Neal, Paraskevi Seferidi

**Affiliations:** 1 Department of Epidemiology and Biostatistics, School of Public Health, Imperial College London, London, UK; 2 The George Institute for Global Health, Sydney, New South Wales Australia; 3 School of Life and Environmental Sciences, Centre for Integrative Ecology, Deakin University, Melbourne, Victoria Australia; 4 School of Public Health, UNSW Sydney, Sydney, New South Wales Australia; 5 Public Health Policy Evaluation Unit, School of Public Health, Imperial College London, London, UK

**Keywords:** Climate-change policy, Environmental impact

## Abstract

Switching between similar food and beverage products may reduce greenhouse gas emissions (GHGe). Here, using consumer data linked to 23,550 product-specific GHGe values, we estimated annual GHGe attributable to product purchases consumed at home in Australia and calculated reductions from specific switches. Potential changes to mean Health Star Rating, mean energy density and the proportion of ultraprocessed foods purchased were assessed. Approximately 31 million tonnes of GHGe were attributable to products consumed at home in 2019, the three highest contributors of GHGe being ‘meat and meat products’ (49%), ‘dairy’ (17%) and ‘non-alcoholic beverages’ (16%). Switching higher-emission products for ‘very similar’ lower-emission products could reduce total emissions by 26%. Switches to ‘less similar’ lower-emission products could lead to a 71% reduction. Switches had little impact on the average Health Star Rating, energy density of purchases and proportion of ultraprocessed foods purchased. Directing manufacturing and marketing towards lower-environmental-impact products and signposting such options to consumers are key.

## Abstract (web summary)

One strategy to reduce the ecological footprint of food systems is to replace higher-emissions food products with lower-emissions alternatives. This study estimates the potential impact of product switches in Australia within ‘very similar’ and ‘less similar’ food categories in terms of greenhouse gas emissions and the nutritional quality and energy density of consumer purchases.

## Main

The food and beverage sector ranks second only to the energy sector in terms of global contributions to carbon dioxide equivalent greenhouse gas emissions (GHGe)^1^. Food and beverage consumption patterns, particularly in higher-income countries, need to change substantially to become environmentally sustainable according to the Paris Climate Agreement and other international reports^2–4^. In these countries, half to three-quarters of consumption is composed of food and beverage products purchased in supermarkets and other grocery retail outlets^5,6^, subsequently making this sector a substantial contributor to overall food and beverage GHGe.

Interventions targeting GHGe attributable to food and beverage products sold in retail stores are limited. The large volume of products available, the insufficient product-specific GHGe data and the complexity of global supply chains all present notable challenges to the development and implementation of effective interventions. Although some existing research describes the environmental impacts of supermarket products^7^, most literature provides GHGe data on broad categories of products and agricultural commodities and no such datasets have been combined with purchase information^8^. Given that supermarket and packaged food and beverage products dominate consumer dietary intake, evidence is needed to inform policy changes (for example, mandatory GHGe labelling of products) that might meaningfully reduce GHGe^9,10^.

As we strive towards sustainable food systems, an important objective is to nudge consumers towards purchasing choices that have lower GHGe^11,12^. The combination of a product’s carbon footprint and its purchase volume will inform its total contribution to food supply GHGe. It is therefore important to understand purchasing patterns of available products in order to develop targeted policies. A recent study modelling GHGe and other environmental impacts of vegan, vegetarian, fish-eating and low- and high-meat-eating diets highlighted the wide range in impacts within each diet type^13^. Across numerous studies, meat and confectionery product types were found to have marginally higher GHGe per serving, though there was also a wide range in product-specific impacts^7,8,14,15^. Other recent studies realize the potential for feasible product switches to substantially contribute to the reduction in food system emissions required to meet the Paris Climate Agreement goals or achieve net zero for the sector^4,16^. Ultimately, environmental food policy should promote products that minimize attributable GHGe and other environmental impacts while also ensuring that health policy targeting better nutrition is not compromised^17^.

In this Analysis, we aimed to utilize product-specific data on the GHGe of Australian food and beverage products (hereon referred to as products) to estimate the potential impact of switching higher-emission products to lower-emission products, within very similar (for example, higher-GHGe white breads switched for lower-GHGe white breads) and less similar categories (for example, higher-GHGe garlic breads to lower-GHGe white breads). We also sought to compare emissions attributable to product purchases across socio-economic groups and quantify the impact of switches on the average nutrient profiling score, level of processing and energy density of purchases.

## Results

Grocery purchase information was used from the 2019 NielsenIQ Homescan Consumer Panel, a dataset that captures all product purchases consumed at home by participating Australian households between January and December each year. There were complete data available from 7,535 households that recorded purchases of a total of 14,957,694 products in 2019 (Supplementary Fig 1). These data were weighted to represent the total Australian population of 25 million people, with an average of 2.58 (standard deviation (s.d.) 1.42) people per household (Table 1a).

**Table 1:** Household and product purchasing characteristics. **a**,**b**, These plots illustrate Australian households and the attributable GHGe (**a**) and volume, nutrient profile, level of processing and energy of foods and beverages brought into the home (**b**), overall and by households grouped according to socio-economic position. GHGe are portrayed in terms of carbon dioxide equivalents, attributable to food and beverage purchases brought into the home

(a)					
NielsenIQ quintiles based on household IRSAD	Number of people (*n*)	Population represented^a^ (*n*)	Mean number of people in household (*n*)	Total GHGe^b^ (million tonnes CO_2_eq per annum)	Mean GHGe^b^ (tonnes CO_2_eq per person per annum)
**Most disadvantaged**					
**Quintile 1**	3,826	4,605,682	2.49 (1.46)	6.52	1.60 (1.05)
**Quintile 2**	3,905	5,045,178	2.60 (1.42)	6.77	1.53 (1.21)
**Quintile 3**	4,030	5,097,491	2.69 (1.47)	6.46	1.44 (0.97)
**Quintile 4**	4,037	5,333,043	2.64 (1.38)	6.22	1.32 (0.94)
**Quintile 5**	3,692	4,922,338	2.50 (1.36)	5.35	1.20 (0.82)
**Least disadvantaged**					
**Total**		**25,003,732**	**2.58 (1.42)**	**31.3**	**1.41 (1.02)**

(b)				
NielsenIQ quintiles based on household IRSAD	Volume of purchases^b^ (mean kg per person per annum)	Nutrient profile of purchases (mean HSR)	Level of processing of purchases (% UPF)	Energy density of purchases^b^ (mean kJ per 100 g)
**Most disadvantaged**				
**Quintile 1**	344.0 (191.6)	3.58 (0.41)	44.3%	723.4 (140.9)
**Quintile 2**	321.7 (180.1)	3.64 (0.41)	45.7%	716.3 (144.7)
**Quintile 3**	316.4 (186.2)	3.66 (0.42)	45.9%	709.6 (145.0)
**Quintile 4**	298.3 (181.1)	3.73 (0.42)	47.5%	696.5 (148.3)
**Quintile 5**	286.1 (170.5)	3.80 (0.43)	49.2%	681.5 (150.5)
**Least disadvantaged**				
**Total**	**312.8 (183.0)**	**3.66 (0.42)**	**46.5%**	**705.1 (146.8)**

^a^The data represented are weighted means (standard deviation) from the NielsenIQ Homescan sample to the Australian population.

^b^There is a statistically significant difference in values across quintiles based on IRSAD tested using ANOVA, where a two-sided *P* value <0.05 was considered statistically significant.

There were 64,041 different products with unique barcodes included in the NielsenIQ Homescan database, and 22,264 (34.8%) were matched to GHGe data in the 2019 FoodSwitch database. The FoodSwitch database contains product-specific nutritional information^18,19^ including a product’s nutrient profiling score based on the Health Star Rating (HSR; a front-of-pack summary score developed by the Australian and New Zealand Governments that assigns products between 0.5 stars (least optimal nutrient profile) and 5.0 stars (most optimal nutrient profile) based on a range of nutritional metrics)^20^, level of processing based on the NOVA classification (NOVA; a system that categorizes food and beverage products to one of four groups based on extent and purpose of processing)^21,22^ and carbon-dioxide-equivalent GHGe estimates^23^ for barcoded Australian products. These products accounted for 88.5% of total units purchased (Supplementary Table 1). Products included in this study were grouped according to the hierarchical FoodSwitch categorization structure comprising 16 major food categories (for example, ‘bread and bakery products’), 74 minor food categories (for example, ‘bread’) and 703 leaf categories (for example, ‘white bread’). The 41,777 products in the NielsenIQ Homescan database that could not be matched to a FoodSwitch product were distributed approximately evenly across different food and beverage categories, so were consequently omitted from analyses. NielsenIQ categories with the largest number of non-matched products were biscuits (5.00% of non-matched products), breads (4.81%) and snack foods (4.77%).

### Estimated GHGe in 2019

Based on the matched purchases, there were 31.3 million (M) tonnes of GHGe attributable to products consumed at home in Australia in 2019. The mean GHGe per person per annum was 1.41 (1.02) tonnes, and the mean GHGe per household per annum was 3.23 (2.24) tonnes (Table 1a). Households in areas with the greatest disadvantage, defined on the basis of postcode using the Index of Relative Social Advantage and Disadvantage (IRSAD)^24,25^, tended to purchase a higher volume of grocery retail products per person per year and therefore contributed significantly higher GHGe per person per annum from this source (*P* value <0.05). In addition, these households purchased products with significantly higher average energy density than households in areas with the least disadvantage (Table 1b; *P* value <0.05). Average HSR did not vary across household socio-economic position (*P* value 0.38).

**Fig. 1:**
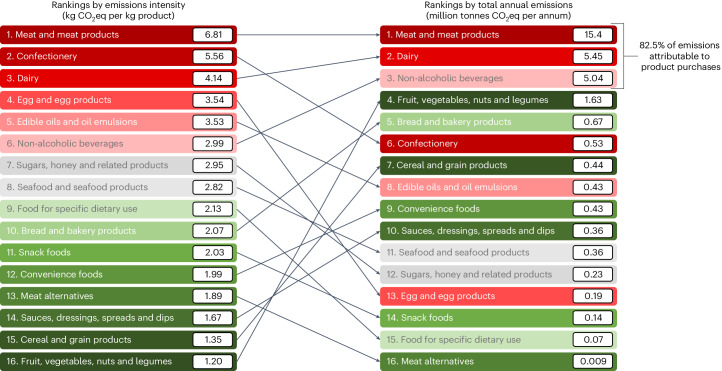
Ranking of major food categories. The illustration compares rankings by emissions intensity and total emissions attributable to foods and beverages brought into Australian homes in 2019. Emission intensity data have been published previously^23^.

**Fig. 2:**
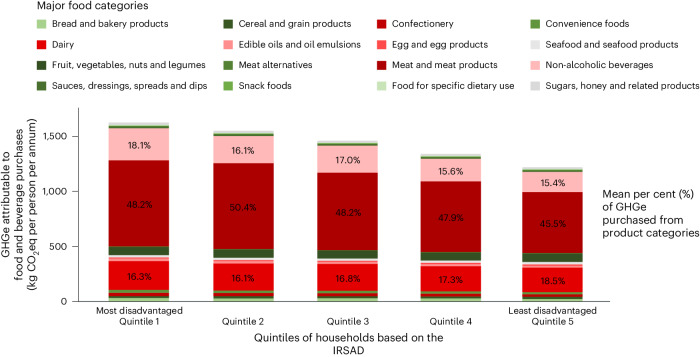
Average GHGe for foods and beverages. This graph illustrates GHGe representative of products brought into the home per person in Australia in 2019 according to quintiles of socio-economic position. Shading and per cent values indicate proportions of GHGe attributable to different food categories. Intake from restaurants and take-away venues are not considered here. For ease of presentation, any category contributing less than <10% of emissions does not have the percentage listed.

The three most GHGe-intensive food categories per kilogram of product were ‘meat and meat products’ (6.81 kg GHGe per kilogram), ‘confectionery’ (5.56 kg GHGe per kilogram) and ‘dairy’ (4.14 kg GHGe per kilogram) (Fig. 1). After weighting for purchase volumes, the food categories that contributed the most to emissions in 2019 were ‘meat and meat products’ (15.4M tonnes GHGe), ‘dairy’ (5.45M tonnes GHGe) and ‘non-alcoholic beverages’ (5.04M tonnes GHGe; Fig. 1). ‘Meat and meat products’ made up 49.0% of total emissions with only 11.2% of total purchases. By contrast, ‘dairy’ and ‘non-alcoholic beverages’, which were ranked third and sixth in terms of kilograms of GHGe per kilogram of product, contributed the second and third largest proportions of total emissions (17.4% and 16.1%, respectively). This was a consequence of their high purchase volumes (21.1% and 20.9% of total purchases, respectively; Supplementary Table 2). The predominant contribution of ‘meat and meat products’, ‘dairy’ and ‘non-alcoholic beverages’ to total GHGe in 2019 was apparent not just for the overall population but also for every socio-economic group (Fig. 2).

### Effects of switching product purchases to reduce GHGe

**Fig. 3:**
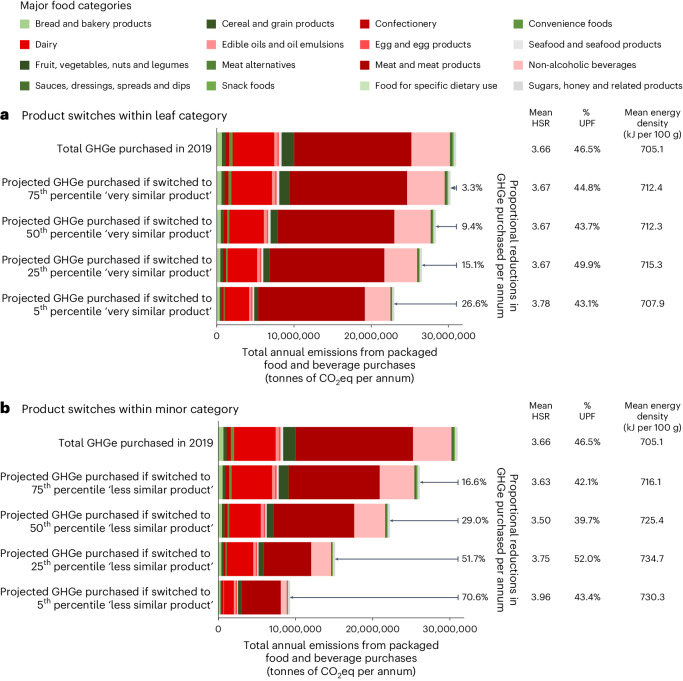
Potential overall reductions in GHGe. **a**,**b**, The graphs illustrate potential reductions in GHGe attributable to foods and beverages brought into the home in Australia in 2019 by making switches to alternative ‘very similar’ products (**a**) and to alternative ‘less similar’ products (**b**). The shading indicates proportions of GHGe attributable to different food categories. The HSR is a score from 0.5 stars (least optimal nutritional profile) to 5.0 stars (most optimal nutritional profile). UPFs are based on the NOVA classification, a system that classifies products as group 1 (minimally processed), group 2 (culinary ingredient), group 3 (processed) or group 4 (ultraprocessed).

Switching higher-GHGe products for similar lower-GHGe products showed clear potential for reducing the GHGe attributable to products brought into the home (example product categories presented in Supplementary Table 3 and example product switches presented in Supplementary Table 4). For the switches made within the 703 leaf categories, where switched products were very similar to those initially selected, we found potential to decrease total emissions by 26.6% when all products above the 5th percentile of GHGe were replaced; the decrease was only 3.3% when products above the 75th percentile were replaced (Fig. 3a). For less similar switches within the minor food category, the potential reduction in GHGe was almost three times greater (70.6% and 16.6% when replacing products above the 5th and 75th percentiles, respectively; Fig. 3b). In general, the greatest absolute and proportional reductions in GHGe all derived from switches made in ‘meat and meat products’, ‘dairy’ and ‘non-alcoholic beverages’ categories (Supplementary Tables 5 and 6).

Switching purchases to products with lower GHGe had only a small impact on the average HSR and proportion of UPFs of purchased products. For example, the mean HSR of products purchased changed from 3.66 to 3.96 and portion of ultraprocessed foods (UPFs) purchased changed from 46.5% to 43.4% when products with GHGe estimates above the fifth percentile were replaced with less similar lower-emission products (Fig. 3b). Likewise, there was little impact on the average energy density of purchases when switching to products with lower GHGe (Fig. 3a,b).

## Discussion

The GHGe attributable to grocery retail purchases in Australia could be greatly reduced by making switches from higher- to lower-emission products. While a modest reduction in GHGe is possible when switching purchases to very similar products, a larger reduction in GHGe could be achieved if switches were made to less similar products within product categories. The plausibility of switching purchases to very similar products is likely to be high, but the consumer acceptability of more substantive switches to less similar products may be lower. The ability to compare products within categories and estimate the potential to reduce annual GHGe from product purchases consumed at home illustrated in these analyses provides a unique opportunity to strengthen Australia’s efforts to reduce national GHGe.

A key finding from the study is that ‘meat and meat products’ contributed to almost 50% of all GHGe attributable to product purchases consumed at home in Australia in 2019, despite accounting for only 11.2% of purchases. The high contribution of ‘meat and meat products’ to the attributable GHGe can be explained by the very high GHGe per kilogram^8,14,15^. ‘Dairy’ and ‘confectionery’, ranked second and sixth, respectively, were leading contributors of attributable GHGe, driven by their high intensity of emissions per kilogram of product. While ‘dairy’ products are often good sources of nutrients, targeted switching within the category could lead to lower emission purchases. As ‘confectionery’ products are all discretionary, encouraging a lower volume of intake would be most beneficial for this category^26^. ‘Non-alcoholic beverages’, although ranked sixth for emissions intensity, were ranked third by total annual emissions weighted by purchases as a result of their high purchase volumes. The top three product categories are most important to address, as they make up 82.5% of total annual food and beverage emissions weighted by purchases. ‘Fruit, vegetables, nuts and legumes’ have very low GHGe per kilogram of product, so while they are the fourth highest contributor to total GHGe because they make up over one-quarter of total purchases, this amounts to only 5.20% of total attributable emissions.

This study found that households with greater area-level disadvantage tended to bring higher volumes of food and beverage purchases into the home, which is consistent with prior research^27^ and corresponds with their higher contribution of attributable GHGe. This is probably because these households tend to purchase a higher proportion of their consumed foods from supermarkets and grocery outlets^28^. Overall, our research found that patterns of consumption, based on the proportion of products purchased per food category, were homogeneous across different household socio-economic positions.

Our study showed that switches made to reduce the GHGe of product purchases did not have a major impact on the average HSR, energy density or proportion of UPFs of product purchases. Previous analyses have reported significant associations between nutrient and energy content, as well as level of processing, and environmental sustainability^29–32^. However, these have been typically comparing between broad product categories such as meat versus dairy versus bread, where differences in both nutrient profiles and sustainability are likely to be large and easily documented^29–32^. Our study explored product switches within very similar categories of foods, which were inherently more likely to result in homogeneity of nutrient profiles than homogeneity of environmental sustainability.

International organizations, including the United Nations^33^, recommend that food policies should consider both impacts on human health and environmental sustainability^34,35^, highlighting the need to ensure that any recommendations targeting reduction of environmental impacts of diets do not contradict existing nutrition policy recommendations. To remain consistent with such dual policy recommendations, we used HSR, NOVA classification and energy density to explore potential unintended side effects of food switches for sustainability on diet. HSR was used as it is the nutrient profiling model currently used by the governments of Australia and New Zealand^20^ to regulate nutrition policy targeting packaged food and beverage products, such as front-of-pack nutrition labelling. It is a standardized and replicable model^36^, similar to nutrient profiling models used in other countries^37^. However, HSR does not holistically capture all aspects of product healthiness^20,38^ or consider broader food consumption patterns and lacks robust validation^39–41^. In general, nutrient profiling models are limited due to their reliance on food components relative to other similar foods (for example, amounts of specific nutrients) rather than a holistic assessment of diet^42^. In some cases, nutrient profiling models have been found to contribute to a false impression of healthiness due to the presentation of positive nutrient qualities (for example, low in fat or high in protein), the non-presentation of negative nutrient qualities (for example, high in added sugars) and little consideration of other health impact factors including level of processing, a phenomenon termed ‘health halos’^43,44^. As such, the effects on HSR reported in this paper do not fully reflect the impact of making switches on the healthiness of the diet. Thus, we also explored associations between product switches for sustainability and the proportion of UPFs purchased based on product NOVA classification. UPF consumption has been repeatedly associated with adverse health outcomes, such as increased risk of obesity, cardio-metabolic outcomes and mortality^45,46^, and the use of the NOVA classification has been shown to improve validity of nutrition classification schemes^47^. By including diverse indicators of diet in our analysis, both currently implemented policy tools, such as HSR, and more holistic classifications, such as NOVA, we provide a rounded overview of the potential impacts of modelled switches on diet, reinforcing the significance of dual action policies and the move away from siloed approaches to food policy^34^.

The joint impact of emissions intensity and purchase volumes on total attributable GHGe highlights the need for urgent policy interventions that target both the most environmentally intensive and the most widely purchased food and beverage products. We found that there is potential for product switches to notably contribute to the reduction in food system emissions required to meet the Paris Climate Agreement goals or achieve net zero for the sector^4^. Further, in all assumed emission-reduction scenarios, most of the potential reduction in GHGe was attributable to three food and beverage categories (that is, ‘meat and meat products’, ‘dairy’ and ‘non-alcoholic beverages’), which identifies them as primary intervention targets. Since total GHGe due to ‘fruit, vegetables, nuts and legumes’ were low compared with the leading three causes of GHGe and their average nutritional quality was high, environmental gains from targeting this and other categories making lesser contributions would be limited. We found only minor variations with respect to purchases of the leading GHGe-contributing food categories for households of different socio-economic position. As such, while the implementation of interventions seeking to reduce GHGe may need to be tailored to suit different socio-economic groups, the target categories for policy intervention (that is, ‘meat and meat products’, ‘dairy’ and ‘non-alcoholic beverages’) should be the same across communities.

Policy intervention options to promote better-for-environment products are not yet being implemented at the industry or consumer level in Australia. In the first instance, Australian policymakers could include the environmental impacts of products into the Australian Dietary Guidelines, following the example of New Zealand^48^. There is also the option to require on-pack labelling of GHGe, similar to the current implementation of the country-of-origin label^49^. While some private carbon-labelling schemes have been implemented on a few products^50,51^, the work here would enable broader labelling implementation in the Australian context that could greatly enhance consumer decision-making^52,53^. Though potentially the least feasible, taxes could be applied to higher-emission and lower-nutritional-quality products using category-specific limits, as this would promote products that are better for people and the planet, and allow accessible dietary variety across households from different socio-economic positions^54^.

These analyses focused on the effects that food and beverage choices could have on reducing GHGe attributable to household product purchases and understanding the potential to optimize product choices based on environmental sustainability. A different method called substitution analysis, which constrains switches by nutrients, volume and energy, could also be done^55,56^, though the minimal differences in the average HSR, energy density and proportion of UPFs purchased of switched products from current purchase patterns suggest that the conclusions from such analyses might be similar. It is also important to note that we did not estimate the potential impacts of product switches on (1) costs, either overall or relative to the purchasing capacity of different socio-economic positions, as the available price data were limited^57^, or (2) other environmental factors (for example, land degradation, scarcity-weighted water use and eutrophication), as these are not yet included in FoodSwitch^23^. While also out of scope for this analysis, future assessment of GHGe reductions by incorporating the frequency of specific product purchases could help to improve both the consumer acceptability and likelihood of product switches. Switching between relevant product categories (for example, ‘meat and meat products’ to ‘meat alternatives’) would be an important next step, as well. These should be considered in future work to enable stakeholders and policymakers to incorporate these impacts into product and policy enhancements. Finally, it will be important to assess the social acceptability of product switches using surveys and behavioural analyses.

This work used product-specific GHGe estimates and household-level purchasing information for an entire food and beverage supply, which provided a unique opportunity to compare emissions both between and within product categories. The NielsenIQ Homescan data are widely considered to provide a valid estimation of products consumed at home, and analyses based on these data have received substantial attention from academia and policymakers^58–60^. They are nationally representative of Australian households by region and socio-economic position and cover almost 90% of total product units purchased yearly. The underlying purchasing data employed here have been used previously to understand the potential health impacts of optimizing nutrient intakes through interventions, such as salt reduction or the implementation of dietary guidelines^61,62^. Both the NielsenIQ Homescan and FoodSwitch datasets have been used in multiple prior analyses of product purchasing patterns in Australia and other jurisdictions^58,63–65^.

There are limitations to the FoodSwitch database, including incomplete coverage of all products sold in Australia and the requirement for imputation of data that are not reported on pack^18^. The product-specific GHGe estimates use a novel methodology that provides summary results that are well aligned with prior reports^7,8^, but there is probably imprecision in the estimates for some individual products. The GHGe estimates include approximations for most ingredients and crudely account for GHGe associated with processing and transport. So far, the estimates do not include geospatial emissions data (that is, global GHGe averages for each ingredient are used), as the source information pertaining to each ingredient is not often available for packaged products. The GHGe associated with refrigeration, packaging and required preparations (for example, cooking or microwaving) are also not currently considered in product estimates.

There are some well-established limitations to the use of NielsenIQ Homescan data for these types of analysis. The absence of information relating to products consumed outside the home (for example, from restaurants and take-away outlets) may lead to an under-estimation of emissions attributable to products purchased across Australia, especially among the least-deprived households. The misreporting of product purchases (for example, omission of products purchased but never brought into the home) may also lead to an overall under-estimation of product emissions across households. Finally, household-specific extrapolation from product purchases to consumption may be miscredited, as purchased products could be shared with, donated or otherwise consumed by persons outside of the household.

In conclusion, large reductions in GHGe attributable to foods and beverages could be achieved through relatively plausible switches in product purchases. There is a clear and immediate opportunity for the Australian government, which is being urged to consider environmental sustainability of diets^66^, to implement policies that nudge consumers and industry towards lower-emission product choices. ‘Meat and meat products’, ‘dairy’ and ‘non-alcoholic beverages’ were identified as the leading causes of GHGe attributable to product purchases consumed at home in Australia and are the three categories where there is clear potential to substantially reduce GHGe^12,62^. Intervention options are multiple, and potential priority actions include a requirement for on-pack labelling of GHGe and the exploration of fiscal incentives that will drive industry production practices and consumer purchasing patterns towards more environmentally friendly choices.

## Methods

In this cross-sectional study, we calculated the annual GHGe attributable to major categories of products purchased from grocery retail outlets and brought into Australian homes in 2019. The study describes variations in food purchases across households of different socio-economic position and ranks the relative contribution of food categories to household GHGe attributable to product purchases. The project was approved by the University of New South Wales Human Research Ethics Committee (approval number HC200244).

### Study population

The NielsenIQ Homescan panel includes about 10,000 households that are surveyed to be representative of the demographic composition and geographic distribution of the Australian population. Data are collected by householders using handheld electronic scanners to record the barcodes of products purchased from all retail outlet types and brought into the home (that is, does not include items that are eaten elsewhere)^27,59,60^. In addition, 284 types of individual products without a barcode (such as unpackaged fruits and vegetables) are recorded by householders scanning the applicable barcode from a list provided by NielsenIQ in booklet form.

The dataset contains information on the characteristics of the households, including the number of household members, number of children and area-level socio-economic position. For assessment across socio-economic positions, the IRSAD system ranks geographic areas using a range of indicators including education, income, occupation and housing. Using the information collected in the latest Australian Bureau of Statistics Household Income and Wealth survey^25^, household areas were divided into quintiles, with quintile 1 representing the most disadvantaged and quintile 5 representing the least disadvantaged.

### Food and beverage data

The 2019 Australian FoodSwitch Annual database used in this analysis contains packaged food and beverage data that have been described in detail previously^18^. Data for the 23,550 included products were obtained by trained data collectors who visited five major supermarket chains in metropolitan Sydney (Coles, Woolworths, IGA, Aldi and Harris Farm) between August and November 2019. Standard and rigorous checks are applied across all products to ensure data quality^67^. Products are organized into main categories (for example, ‘meat and meat products’), then finer minor categories (for example, ‘processed meat’) and subcategories (for example, ‘meat pies’ and ‘coated/breaded frozen/chilled meat’). These categories were derived from a system originally developed by the Global Food Monitoring Group^67^. Products from 16 major food and beverage categories were included (Supplementary Table 3). Products from the ‘alcoholic beverages’ and ‘vitamins and supplements’ categories were not included in the current analysis because they are not included in the scope of the HSR.

The NielsenIQ Homescan purchase data were linked to the 2019 FoodSwitch database in order to assess product nutrition information, HSR, NOVA and GHGe. The product-specific GHGe values were estimated primarily on the basis of ingredient life cycle assessment data, using the cradle-to-farm gate system boundary, and adjusted for ingredient processing and ingredient refuse. The ingredient GHGe values were then weighted by ingredient proportions and summed to estimate the product-specific GHGe value, with additional adjustments applied to account for product processing and transport^23^.

Products were matched in a three-step process. First, we aimed to match as many products as possible by their unique barcodes. Products without barcode information were matched according to product name using previously described methods^27,68^. We then matched products using their exact product name, and finally using their product name after removing any nutritionally irrelevant descriptors (for example, container type or product shape). The 284 unpackaged, unbarcoded NielsenIQ products were assigned the average GHGe for the closest corresponding leaf category in the FoodSwitch database.

### Outcomes

The primary outcome for these analyses was the estimated tonnes of GHGe attributable to product purchases consumed at home in Australia in 2019. This was estimated by totalling the GHGe for each matched product based on the quantity (weight) purchased over the year to obtain an annual GHGe value for product purchases made by each household for each product. The secondary outcomes were the average nutrient profiling score as defined by the mean HSR, the mean energy density and proportion of UPFs purchased as defined by the NOVA group 4 classification, recorded in kJ per 100 g, of products purchased.

### Statistical analysis

All results were weighted to represent the overall Australian population using survey sample weights based on the latest Australian Bureau of Statistics census data provided by NielsenIQ Homescan^24^. To control for under-reporting of purchases, households were included if they recorded at least one barcode per week for at least 50% of the 52-week period and spent an average of at least $5 per week on product purchases. We described characteristics and purchasing patterns of included households both for the overall population and across quintiles based on the IRSAD. Differences across quintiles were assessed using one-way analysis of variance (ANOVA). We also described the GHGe contribution of purchases across the 16 major food categories by socio-economic position.

We ranked each of the major food categories, first according to emissions per kilogram of product (that is, kilogram GHGe per kilogram of product) and then according to the total emissions attributable to annual purchases of that product (that is, tonnes GHGe purchased per year). This allowed us to explore the changes in ranking of product categories when weighted by purchases.

We tested the potential impact on GHGe, mean HSR, mean energy density (in kJ per 100 g) and proportion of UPFs purchased (%) of switching product purchases in two ways. First, we examined the impact of making small changes to initial purchasing patterns by switching products within leaf categories (*n* = 703), which include very similar products that could be easily switched and are most similar in the functional sense, such as use and contribution to the diet (for example, higher-GHGe white bread products switched for lower-GHGe white bread products). See Supplementary Table 3 for more examples. To estimate the effect of product switching, we determined the 75th percentile GHGe value within each leaf category. We then replaced the GHGe, HSR, energy density values and NOVA group classification of all purchased products with a GHGe value above the 75th percentile with the GHGe, HSR, energy density values and NOVA group classification of the product at the 75th percentile. In turn, we repeated this process for the products lying above the 50th, 25th and 5th percentiles of GHGe to test the impact of progressively switching purchases towards those products with the lowest GHGe^4,29^. Next, we repeated this process to examine the potential impact of switching products within the broader minor food categories (*n* = 74) of less similar products that maintain less but still some likeliness in functionality (for example, from ‘garlic bread’ products to ‘white bread’ products; see Supplementary Table 3 for more examples). Potential impacts are presented overall and by the 16 major food categories.

All data preparation and statistical analyses were conducted using Stata BE 17 (Stata Corp) and Excel. A two-sided *P* value <0.05 was considered statistically significant.

### Reporting summary

Further information on research design is available in the Nature Portfolio Reporting Summary linked to this article.

### Supplementary information

Supplementary Information Supplementary Tables 1–6 and Fig. 1.

Reporting Summary

## Data Availability

The Ecoinvent 3.7.1 and Agri-footprint 5.0 databases contained in SimaPro were used to assign ingredient GHGe values. The product-specific source data obtained from the FoodSwitch database are proprietary and sample data can only be made available upon request.

## Code availability

The code will be made available upon reasonable request.
