# Supplementary material for: Switches in food and beverage product purchases can reduce greenhouse gas emissions in Australia
Source: Nat Food. 2024 May 28;5(6):524–32. doi: 10.1038/s43016-024-00971-6 (PMC11199139; doi:10.1038/s43016-024-00971-6)

nature food

Analysis https://doi.org/10.1038/s43016-024-00971-6

Switches in food and beverage product purchases can reduce greenhouse gas emissions in Australia

In the format provided by the authors and unedited

SUPPLEMENTARY MATERIALS

Supplementary Table 1| Success rate for matching food and beverage products between the NielsenIQ and FoodSwitch databases**.** Products were excluded from both the Nielsen Homescan and FoodSwitch databases if they were not relevant for analyses. This included non-food and beverage products, such as cleaning products. Variety packs with multiple NIPs and products were also excluded as these cannot be categorised. The databases were merged by matching products according to barcode (*Match Step 1*) followed by matching by exact product name (*Match Step 2*) and finally by matching product names after removing irrelevant descriptors (e.g., container type or product shape) (*Match Step 3*) in order to optimise the match rate. A majority of unmatched products were found to be unpackaged such as fruits, vegetables, store-prepared bakery items and ready-to-eat dishes which are not required to display the same level of nutrition information as packaged products.

		**Barcodes**		**Units sold**	
**Match Step 1**<br>**Direct match by barcode**	Unmatched to GHGe values	44,557	(69.6%)	2,316,324	(13.7%)
	Matched to GHGe values	19,484	(30.4%)	14,581,046	(86.3%)
	Total	***64,041***		***16,897,370***	
**Match Step 2**<br>**Direct match by product name**	Unmatched to GHGe values	41,819	(65.3%)	1,943,061	(11.5%)
	Matched to GHGe values	22,222	(34.7%)	14,954,309	(88.5%)
	Total	***64,041***		***16,897,370***	
**Match Step 3**<br>**Direct match by removal of nutritionally irrelevant descriptors**	Unmatched to GHGe values	41,777	(65.2%)	1,939,676	(11.5%)
	Matched to GHGe values	22,264	(34.8%)	14,957,694	(88.5%)
	Total	***64,041***		***16,897,370***	

Supplementary Table 2| Proportions of greenhouse gas emissions contributed by supermarket purchases of different food groups across Australian households in 2019

**Major food category**	**Total weight of purchases** *(million tonnes per annum)*		**Estimated total GHGe of purchases** *(million tonnes CO_2eq per annum)*		**Mean weight of purchases** *(kg per person per annum)*	**Mean GHGe of purchases** *(tonnes CO_2eq per person per annum)*
Bread and bakery products	0.39	(5.73%)	0.67	(2.14%)	17.5	0.029
Cereal and grain products	0.29	(4.26%)	0.44	(1.40%)	12.4	0.018
Confectionery	0.09	(1.27%)	0.53	(1.70%)	4.23	0.026
Convenience foods	0.12	(1.79%)	0.43	(1.38%)	6.21	0.021
Dairy	1.44	(21.1%)	5.45	(17.4%)	64.5	0.242
Edible oils and oil emulsions	0.11	(1.66%)	0.43	(1.38%)	5.07	0.019
Egg and egg products	0.05	(0.77%)	0.19	(0.60%)	2.41	0.009
Food for specific dietary use	0.03	(0.45%)	0.07	(0.21%)	1.26	0.003
Fruit, vegetables, nuts and legumes	1.70	(25.0%)	1.63	(5.20%)	79.3	0.076
Meat alternatives	0.004	(0.07%)	0.009	(0.03%)	0.22	0.0004
Meat and meat products	0.76	(11.2%)	15.4	(49.0%)	34.1	0.684
Non-alcoholic beverages	1.43	(20.9%)	5.04	(16.1%)	67.4	0.236
Sauces, dressings, spreads and dips	0.19	(2.83%)	0.36	(1.15%)	8.64	0.015
Seafood and seafood products	0.07	(1.08%)	0.36	(1.14%)	3.52	0.016
Snack foods	0.06	(0.88%)	0.14	(0.44%)	2.61	0.006
Sugars, honey and related products	0.07	(1.09%)	0.23	(0.73%)	3.39	0.010
Total	***6.82***		***31.3***		***19.6***	***0.088***

Supplemental Table 3| Example product minor categories (representing less similar switches) and product leaf categories (representing very similar switches)

**Major Category - Bread and bakery products**			
Minor Category - Bread			
Leaf Categories	Bagels	Naan	Regular flat bread
	Bread rolls	Other grain breads not otherwise specified	Roti
	Croutons	Other plain bread not otherwise specified	Rye bread
	Crumpets	Other savoury bread not otherwise specified	Soy & linseed bread
	English muffins	Other sweet bread not otherwise specified	Taco shells
	Fruit bread	Pappudoms	Tortillas
	Garlic bread	Pita bread	White bread
	Mixed grain sourdough	Pizza bases	Wholemeal bread
	Multigrain bread	Plain ice cream cones	Regular flat bread

**Major Category - Meat and meat products**			
Minor Category - Processed meat			
Leaf Categories	Bacon	Dried meat	Pork sausages
	Beef burgers	Hotdogs	Salami
	Beef sausages	Kangaroo sausages	Sausage rolls
	Cabanossi and twiggy sticks	Kebabs	Sliced beef
	Canned beef	Kransky	Sliced chicken
	Canned chicken	Lamb sausages	Sliced ham
	Canned ham (e.g. SPAM)	Meat pies	Sliced luncheon meat
	Canned other meat not otherwise specified	Other meat burgers not otherwise specified	Sliced pork
	Chicken burgers	Other meat with pastry not otherwise specified	Sliced turkey
	Chicken sausages	Pancetta and prosciutto	Uncoated frozen/chilled processed meat
	Chorizo	Pate and meat spreads	Whole hams and similar products
	Coated/breaded frozen/chilled meat	Polish salami	

Supplementary Table 4| Examples of less similar switches for products in 12 of the major food categories. Leaf categories are noted in *italics* in each cell above the product names. Brand names have been removed for anonymity of data.

Major category	Minor category	Original product	75th percentile switch	50th percentile switch	25th percentile switch	5th percentile switch
**Bread and bakery products**	**Bread**	*Other sweet bread not otherwise specified*<br>Chocolate Chip Brioche Rolls	*Regular flat bread*<br>Mixed Grain Wraps	*White bread*<br>Sliced White Bread	*Bread rolls*<br>Dinner Rolls	*White bread*<br>Sourdough Loaf
**Cereal and grain products**	**Breakfast cereals**	*Breakfast cookies/rusks etc.*<br>Breakfast Chocolate Biscuits	*Corn flakes*<br>Corn Flakes	*Breakfast bites*<br>Crunchy Honey Bran Bite	*Flavoured oats*<br>Almond Pecan and Hazelnut Porridge Sachet	*Plain oats*<br>Quick Oats
**Confectionery**	**Chocolate and sweets**	*Block dark chocolate (no nuts)*<br>Baking 70% Cocoa Dark Chocolate	*Block dark chocolate (no nuts)*<br>Dark Chocolate Peppermint Block	*Single chocolate bars*<br>Original Chocolate Bar	*Chocolate bars in packs*<br>Crunch Chocolate Bar	*Mints*<br>Chewy Dragees Mint Flavour
**Convenience foods**	**Soup**	*Canned other soup with meat*<br>Beef & Veg Soup	*Canned pea and ham soup*<br>Pea & Ham Soup	*Other soup with veg dry mix*<br>Dutch Curry & Rice Soup	*Chilled other soup with veg*<br>Curried Sweet Potato & Turmeric Soup	*Canned other soup*<br>All Natural Three Bean Chunky Soup

Major category	Minor category	Original product	75th percentile switch	50th percentile switch	25th percentile switch	5th percentile switch
**Dairy**	**Yoghurt and yoghurt drinks**	*Yoghurt with muesli or other non-fruit additions*	*Apple-based yoghurt*	*Strawberry yoghurt*	*Drinking yoghurt*	*Non-dairy yoghurts*
		Sweetened Greek Style Strained Yogurt with Honey Granola	Dairy Apple & Rhubarb Twist Yogurt	Strawberry Greek Style Yogurt	Strawberry Yoghurt Drink	Gluten Free Cultured Coconut Milk with Vanilla Yoghurt
**Fruit, vegetables, nuts and legumes**	**Vegetables**	*Plain frozen vegetables*	*Peppers/capsicum*	*Other canned vegetables not otherwise specified*	*Pickles/gherkins*	*Other canned vegetables not otherwise specified*
		Peas & Carrots	Capsicums	Baby Carisma Potatoes	Gherkins	Onions
**Meat alternatives**	**Meat alternatives**	*Other meat-free products not otherwise specified*	*Plain tofu*	*Other meat-free products not otherwise specified*	*Plain tofu*	*Meat-free burgers*
		Textured Vegetable Protein	Organic Tofu Hi-Protein Firm	Savoury Vegie Mince	Silken Tofu	Black Bean Beetroot Burger
**Meat and meat products**	**Processed meat**	*Dried meat*	*Meat pies*	*Kransky*	*Sliced ham*	*Coated/breaded frozen/chilled meat*
		Mini-Stick Biltong Peri Peri	Beef with Caramelised Onion & Cabernet Sauvignon	Traditional Kransky	Triple Smoked Ham	Chicken Breast Tenders Southern Style

Major category	Minor category	Original product	75th percentile switch	50th percentile switch	25th percentile switch	5th percentile switch
**Non-alcoholic beverages**	**Soft Drinks**	*Cola sugar-free*	*Sugar-sweetened cola*	*Other sugar-sweetened soft drinks not otherwise specified*	*Other sugar-sweetened soft drinks not otherwise specified*	*Ice tea drinks*
		Sugar-free Vanilla Cola	Original Cola	Dry Ginger Ale Soft Drink	Raspberry Soft Drink	Organic Kombucha Ginger Lemon
**Sauces, dressings, spreads and dips**	**Spreads and dips**	*Seafood-based dips*	*Hummus*	*Smooth peanut butter*	*Spinach dips*	*Salsa*
		Prawn Seafood Dip with a Hint of Lemon	Hummus Dip	Protein Peanut Butter Dip	Gluten Free Classic Spinach Dip	Chunky Tomato Salsa Mild
**Seafood and seafood products**	**Seafood**	*Other canned fish not otherwise specified*	*Coated/battered frozen fish*	*Chilled raw fish flavoured*	*Anchovies*	*Plain canned sardines*
		Skinless & Boneless Rainbow Trout Smoked Fillets	Panko Squid Strips	Marinated Salmon Mustard & Dill Sauce	Anchovies Fillets In Olive Oil	Sardines In Extra Virgin Olive Oil
**Snack foods**	**Crisps and snacks**	*Chicken flavour potato crisps*	*Corn chips*	*Sweet chilli potato crisps*	*Popcorn*	*Wholegrain chips*
		Roast Chicken Flavour crisps	Roasted Beetroot & Ricotta Tortilla Chips	Sweet Chilli & Sour Cream Flavoured Potato Chips	Natural Popcorn Sea Salted Caramel	Cheese Flavour Whole Grain Bites

Supplementary Table 5| Description of product purchases after making switches to alternative *very similar*, lower-emission products

Major food category	Estimated total GHGe of purchases *(million tonnes CO_2eq per annum)*		Estimated total GHGe of purchases, emissions limited at 5th percentile *(million tonnes CO_2eq per annum)*		Mean HSR		Mean NOVA		Mean energy density *(kJ per 100g of product)*	
					**Original purchases**	**Purchases with emissions limited at 5th percentile**	**Original purchases**	**Purchases with emissions limited at 5th percentile**	**Original purchases**	**Purchases with emissions limited at 5th percentile**
Bread and bakery products	0.67	(2.14%)	0.39	(1.68%)	3.63	3.76	2.42	2.41	724.8	719.0
Cereal and grain products	0.44	(1.40%)	0.24	(1.04%)	3.68	3.81	2.38	2.36	713.2	707.5
Confectionery	0.53	(1.70%)	0.28	(1.21%)	3.58	3.71	2.46	2.44	740.6	735.5
Convenience foods	0.43	(1.38%)	0.13	(0.56%)	3.61	3.73	2.48	2.46	716.4	710.9
Dairy	5.45	(17.4%)	3.17	(13.8%)	3.66	3.79	2.39	2.37	711.2	705.5
Edible oils and oil emulsions	0.43	(1.38%)	0.25	(1.10%)	3.68	3.80	2.35	2.34	709.2	703.9
Egg and egg products	0.19	(0.60%)	0.19	(0.81%)	3.70	3.82	2.35	2.33	701.3	695.4
Food for specific dietary use	0.07	(0.21%)	0.03	(0.15%)	3.72	3.83	2.34	2.32	692.2	686.8
Fruit, vegetables, nuts and legumes	1.63	(5.20%)	0.51	(2.24%)	3.74	3.86	2.31	2.29	689.9	684.1
Meat alternatives	0.009	(0.03%)	0.004	(0.02%)	3.88	3.98	2.30	2.27	664.4	657.2
Meat and meat products	15.4	(49.0%)	13.8	(60.1%)	3.64	3.77	2.39	2.37	712.9	707.7
Non-alcoholic beverages	5.04	(16.1%)	3.32	(14.4%)	3.61	3.74	2.45	2.43	715.5	710.4
Sauces, dressings, spreads and dips	0.36	(1.15%)	0.16	(0.70%)	3.66	3.78	2.39	2.37	709.8	704.8
Seafood and seafood products	0.36	(1.14%)	0.21	(0.92%)	3.59	3.73	2.47	2.45	740.7	735.4
Snack foods	0.14	(0.44%)	0.07	(0.32%)	3.64	3.78	2.49	2.47	716.4	712.0
Sugars, honey and related products	0.23	(0.73%)	0.21	(0.91%)	3.66	3.79	2.36	2.34	715.4	709.6
Total	***31.3***		***23.0***		***3.66***	***3.78***	***2.40***	***2.38***	***705.1***	***707.9***

GHGe = Greenhouse Gas Emissions
HSR = Health Star Rating

Supplementary Table 6| Description of product purchases after making switches to alternative *less similar*, lower-emission products

					Mean HSR		Mean NOVA		Mean energy density *(kJ per 100g of product)*	
**Major food category**	**Estimated total GHGe of purchases** *(million tonnes CO_2eq per annum)*		**Estimated total GHGe of purchases, emissions limited at 5th percentile** *(million tonnes CO_2eq per annum)*		**Original purchases**	**Purchases with emissions limited at 5th percentile**	**Original purchases**	**Purchases with emissions limited at 5th percentile**	**Original purchases**	**Purchases with emissions limited at 5th percentile**
Bread and bakery products	0.67	(2.14%)	0.29	(3.13%)	3.63	3.95	2.43	2.54	724.8	741.5
Cereal and grain products	0.44	(1.40%)	0.19	(2.01%)	3.68	3.98	2.38	2.49	713.2	729.8
Confectionery	0.53	(1.70%)	0.17	(1.82%)	3.58	3.91	2.46	2.57	740.6	756.9
Convenience foods	0.43	(1.38%)	0.09	(0.99%)	3.61	3.92	2.48	2.58	716.4	735.2
Dairy	5.45	(17.4%)	1.27	(13.7%)	3.66	3.96	2.39	2.51	711.2	727.7
Edible oils and oil emulsions	0.43	(1.38%)	0.24	(2.62%)	3.68	3.98	2.35	2.48	709.2	725.8
Egg and egg products	0.19	(0.60%)	0.19	(2.01%)	3.70	3.99	2.35	2.48	701.3	718.7
Food for specific dietary use	0.07	(0.21%)	0.03	(0.35%)	3.72	4.00	2.34	2.46	692.2	710.0
Fruit, vegetables, nuts and legumes	1.63	(5.20%)	0.44	(4.73%)	3.74	4.02	2.31	2.43	689.9	707.2
Meat alternatives	0.009	(0.03%)	0.003	(0.04%)	3.88	4.13	2.30	2.35	664.4	680.3
Meat and meat products	15.4	(49.0%)	5.10	(54.9%)	3.64	3.96	2.39	2.53	712.9	730.0
Non-alcoholic beverages	5.04	(16.1%)	0.79	(8.46%)	3.61	3.93	2.45	2.56	715.5	732.6
Sauces, dressings, spreads and dips	0.36	(1.15%)	0.11	(1.17%)	3.66	3.97	2.39	2.51	709.8	727.0
Seafood and seafood products	0.36	(1.14%)	0.12	(1.28%)	3.59	3.93	2.47	2.58	740.7	756.7
Snack foods	0.14	(0.44%)	0.05	(0.53%)	3.64	3.91	2.49	2.58	716.4	735.9
Sugars, honey and related products	0.23	(0.73%)	0.21	(2.25%)	3.66	3.97	2.36	2.49	715.4	730.9
Total	***31.3***		***9.28***		***3.66***	***3.96***	***2.40***	***2.51***	***705.1***	***730.3***

GHGe = Greenhouse Gas Emissions
HSR = Health Star Rating

Supplementary Figure 1| Flowchart of household data preparation and exclusion criteria. Households were excluded from analyses based on eligibility criteria provided by NielsenIQ. To be included in the analyses, households must have been on the panel for the entire 12-month time frame and reported purchase data (at least one barcode per week) for at least 50% of the weeks. Households' data were deemed unreliable and excluded from analyses if they were missing any demographic information or if thresholds for expenditure were not met (≥$5 on average for each week over the time frame, i.e., at least $260 per household over the 52-week period).

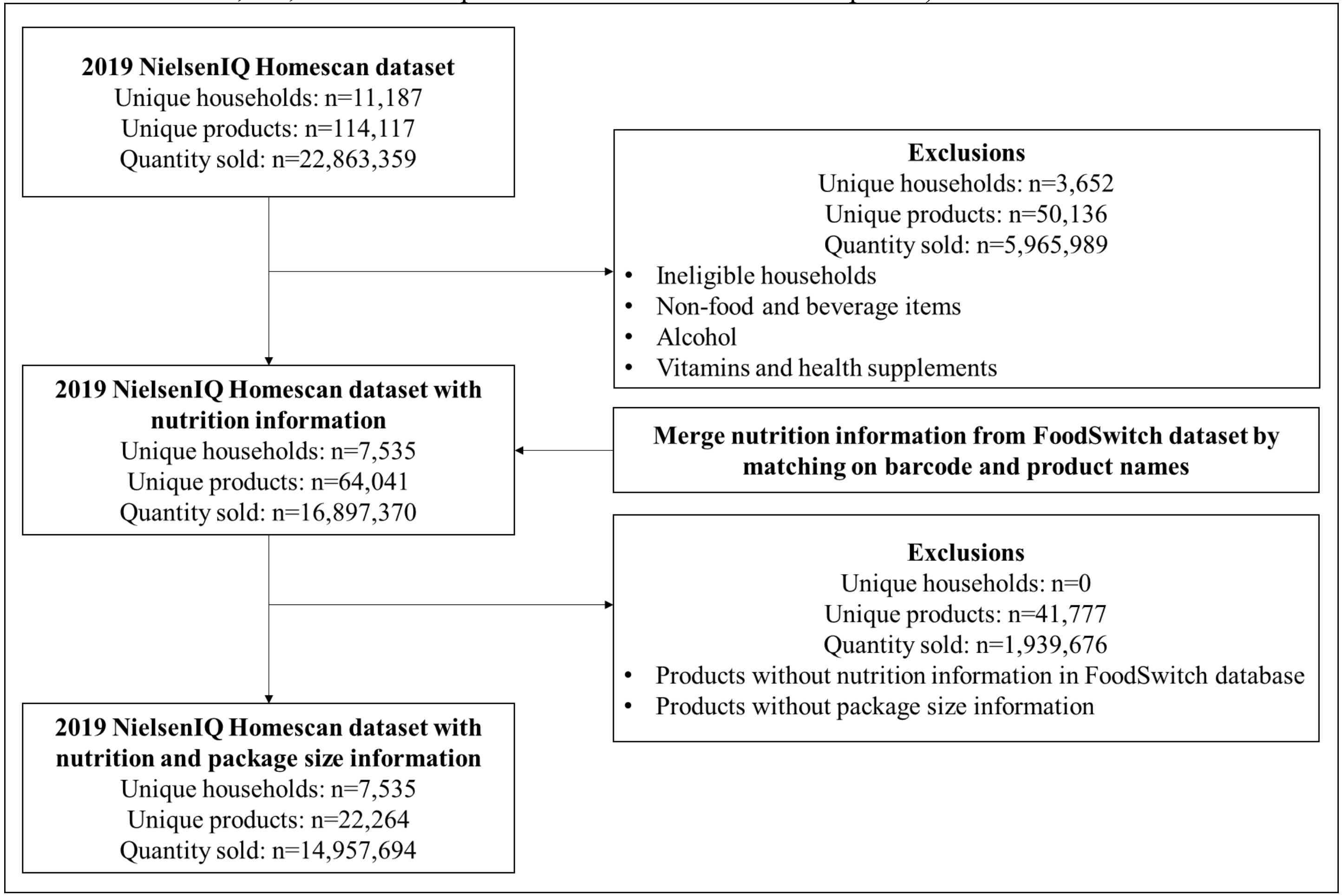

Supplement: Supplementary file 1 — Supplementary Tables 1–6 and Fig. 1. [file 43016_2024_971_MOESM1_ESM.pdf]
