# Supplementary material for: Switches in food and beverage product purchases can reduce greenhouse gas emissions in Australia
Source: Nat Food. 2024 May 28;5(6):524–32. doi: 10.1038/s43016-024-00971-6 (PMC11199139; doi:10.1038/s43016-024-00971-6)
Supplement: Supplementary file 2 — Reporting Summary [file 43016_2024_971_MOESM2_ESM.pdf]

nature portfolio

Corresponding author(s): Allison Gaines

Last updated by author(s): Feb 18, 2023

Reporting Summary

Nature Portfolio wishes to improve the reproducibility of the work that we publish. This form provides structure for consistency and transparency in reporting. For further information on Nature Portfolio policies, see our Editorial Policies and the Editorial Policy Checklist.

Statistics

For all statistical analyses, confirm that the following items are present in the figure legend, table legend, main text, or Methods section.

n/a	Confirmed	
☐	☒	The exact sample size (n) for each experimental group/condition, given as a discrete number and unit of measurement
☒	☐	A statement on whether measurements were taken from distinct samples or whether the same sample was measured repeatedly
☐	☒	The statistical test(s) used AND whether they are one- or two-sided<br>*Only common tests should be described solely by name; describe more complex techniques in the Methods section.*
☐	☒	A description of all covariates tested
☒	☐	A description of any assumptions or corrections, such as tests of normality and adjustment for multiple comparisons
☐	☒	A full description of the statistical parameters including central tendency (e.g. means) or other basic estimates (e.g. regression coefficient) AND variation (e.g. standard deviation) or associated estimates of uncertainty (e.g. confidence intervals)
☐	☒	For null hypothesis testing, the test statistic (e.g. F, t, r) with confidence intervals, effect sizes, degrees of freedom and P value noted<br>*Give P values as exact values whenever suitable.*
☒	☐	For Bayesian analysis, information on the choice of priors and Markov chain Monte Carlo settings
☒	☐	For hierarchical and complex designs, identification of the appropriate level for tests and full reporting of outcomes
☒	☐	Estimates of effect sizes (e.g. Cohen's d, Pearson's r), indicating how they were calculated

Our web collection on statistics for biologists contains articles on many of the points above.

Software and code

Policy information about availability of computer code

Data collection	Data were collected using the FoodSwitch Data Collection phone application.
Data analysis	All data preparation and statistical analyses were conducted using Stata BE 17 (Stata Corp) and Excel.

For manuscripts utilizing custom algorithms or software that are central to the research but not yet described in published literature, software must be made available to editors and reviewers. We strongly encourage code deposition in a community repository (e.g. GitHub). See the Nature Portfolio guidelines for submitting code & software for further information.

Data

Policy information about availability of data

All manuscripts must include a data availability statement. This statement should provide the following information, where applicable:

- Accession codes, unique identifiers, or web links for publicly available datasets
- A description of any restrictions on data availability
- For clinical datasets or third party data, please ensure that the statement adheres to our policy

The Ecoinvent 3.7.1 and Agri-footprint 5.0 databases contained in SimaPro were used to assign ingredient greenhouse gas emission values. The product-specific source data obtained from the FoodSwitch database are proprietary.

Human research participants

Policy information about studies involving human research participants and Sex and Gender in Research.

Reporting on sex and gender	Not applicable.
Population characteristics	See below in "Behavioural & social sciences study design" questions
Recruitment	To ensure the Homescan panel are representative of Australian households, Nielsen has formed geographical segments with quotas for each geographical area to avoid clustering of households by location. They also control for other factors in the recruitment stage that are relevant to grocery purchasing, including household size, lifestage and income level. To ensure recruited households are demographically and geographically representative of Australian households, the data collected by recruited households are projected to the demographics of the Australia population.
Ethics oversight	The project was approved by the University of New South Wales Human Research Ethics Committee (approval number HC200244).

Note that full information on the approval of the study protocol must also be provided in the manuscript.

Field-specific reporting

Please select the one below that is the best fit for your research. If you are not sure, read the appropriate sections before making your selection.

☐ Life sciences ☒ Behavioural & social sciences ☐ Ecological, evolutionary & environmental sciences

For a reference copy of the document with all sections, see nature.com/documents/nr-reporting-summary-flat.pdf

Behavioural & social sciences study design

All studies must disclose on these points even when the disclosure is negative.

Study description	In this mixed-methods cross-sectional study, we calculated the annual GHGe attributable to major categories of products purchased from grocery retail outlets and brought into Australian homes in 2019. The study describes variations in food purchases across households of different socioeconomic position and ranks the relative contribution of food categories to household GHGe attributable to product purchases.
Research sample	The 2019 NielsenIQ Homescan Consumer Panel, the most comprehensive Australian dataset that represents product purchases consumed at home each year
Sampling strategy	Nielsen Homescan maintains a panel of approximately 10,000 households chosen using a stratified sampling procedure. These households are recruited through an online application process and are chosen to ensure representative sociodemographic and economic characteristics.
Data collection	Data are collected by householders using handheld electronic scanners to record the barcodes of products purchased from all retail outlet types and brought into the home. These household members are blinded to the study hypothesis.
Timing	January 1, 2019 to December 31, 2019
Data exclusions	To control for under-reporting of purchases, households were excluded (n=3,652) if they recorded, on average, less than one barcode per week for at least 50% of the 52-week period and spent an average of less than $5 per week on product purchases. Additionally, product purchases were excluded if they could not be matched to a product with a greenhouse gas emission estimate in FoodSwitch (approximately 10% of purchases recorded and brought into the home).
Non-participation	No participants dropped out.
Randomization	Stratified random sampling was used.

Reporting for specific materials, systems and methods

We require information from authors about some types of materials, experimental systems and methods used in many studies. Here, indicate whether each material, system or method listed is relevant to your study. If you are not sure if a list item applies to your research, read the appropriate section before selecting a response.

Materials & experimental systems

n/a	Involved in the study
☒	☐ Antibodies
☒	☐ Eukaryotic cell lines
☒	☐ Palaeontology and archaeology
☒	☐ Animals and other organisms
☒	☐ Clinical data
☒	☐ Dual use research of concern

Methods

n/a	Involved in the study
☒	☐ ChIP-seq
☒	☐ Flow cytometry
☒	☐ MRI-based neuroimaging
